# Physiotherapists’ knowledge about the diagnosis, treatment and physical activity of patients with idiopathic scoliosis

**DOI:** 10.3389/fmed.2024.1414709

**Published:** 2024-10-10

**Authors:** Piotr Wójcik, Anna Wójcik, Paulina Ewertowska, Dariusz Czaprowski

**Affiliations:** ^1^Department of Public Health, Olsztyn University, Olsztyn, Poland; ^2^Center of Body Posture, Olsztyn, Poland; ^3^Department of Physical Culture, Gdansk University of Physical Education and Sport, Gdansk, Poland; ^4^Department of Physiotherapy, School of Public Health, Collegium Medicum, University of Warmia and Mazury, Olsztyn, Poland

**Keywords:** spinal deformity, knowledge, treatment, physiotherapy, SOSORT recommendations

## Abstract

Idiopathic scoliosis (IS) is a three-dimensional spinal deformity with an unknown etiology which, when left untreated, can have negative health consequences. Physiotherapy plays an important role in the treatment of IS, which is why physiotherapists should have adequate theoretical and practical knowledge about IS diagnosis and treatment methods. The aim of this study was to assess physiotherapists’ knowledge about IS, its diagnosis, treatment and recommended physical activities for subjects with IS. The influence of post-graduate courses, workplace, academic degree, work experience, education and membership in associations dealing with spinal deformities was also analyzed. The study involved 116 licensed physiotherapists. The research method was a diagnostic survey, and the research tool was a self-designed questionnaire. The questionnaire which included demographic questions to elicit information about the respondents and questions concerning IS, its diagnosis, treatment and recommended physical activity in the course of IS. The questions were created according to the recommendation of the Society on Scoliosis Orthopaedic and Rehabilitation Treatment. In the questionnaire, the respondents’ knowledge was assessed on the percent scale (100–90% of correct answers – full knowledge, 89–60% of correct answers – good knowledge, 59–50% of correct answers – moderate knowledge, <50% of correct answers – poor knowledge). The physiotherapists correctly answered from 60 to 89% of the questions which meant their good level of knowledge of IS. Factors such as post-graduate courses, workplace and academic degree influenced the knowledge of Polish physiotherapists regarding IS (*p* = 0.003, *p* = 0.036, *p* = 0.05, respectively). Physiotherapists who completed courses based on physiotherapy recommended by SOSORT demonstrated a higher level of knowledge compared to those without postgraduate courses (*p* = 0.018). The physiotherapists who ran the private individual physiotherapy practice demonstrated a higher level of knowledge compared to those working in a hospital (*p* = 0.058). Work experience and education have no influence on the physiotherapists’ knowledge about IS. Polish physiotherapists have a good knowledge of IS. Post-graduate courses (courses on Physiotherapeutic Scoliosis Specific Exercises), the respondents’ place of employment (private individual physiotherapy practice) and academic degree influence the knowledge about IS. Work experience, education and membership in associations dealing with spinal deformities have no influence on the physiotherapists’ knowledge about IS.

## 1 Introduction

Idiopathic scoliosis (IS) is a three-dimensional spinal deformity with an unknown etiology (1–4). The theories regarding the etiopathogenesis of the disorder share the understanding that IS is a multifactorial condition. Idiopathic scoliosis account for around 80–90% of all structural scoliosis. IS affects 2–3% of the adolescent population (4–7). Idiopathic scoliosis can have serious negative consequences such as trunk deformity, worsening of the esthetics, decreased physical capacity, back pain, and, in consequence, decreased quality of life. Physiotherapy plays an important role in the treatment of IS (1, 8, 9), which is why physiotherapists should have adequate theoretical and practical knowledge about IS diagnosis and treatment.

Physiotherapists’ and physiotherapy students’ knowledge about IS diagnosis and treatment has been assessed by researchers in different regions of the world, including the United Kingdom (10), the United States (11), South Africa (12), and Turkey (13). In Poland, so far, the knowledge was verified only once (14) on a small group of 37 third-year physiotherapy students of the Medical University of Silesia. All of the cited authors concluded that the surveyed respondents had inadequate knowledge about IS, and its diagnosis and treatment. These results indicate that physiotherapists’ knowledge about IS requires further improvement.

The aim of this study was to assess physiotherapists’ knowledge about IS, its diagnosis and treatment, as well as the recommended physical activities for subjects with IS. Factors that could influence the respondents’ knowledge, including post-graduate training, place of employment, academic degree, work experience, education and membership in associations dealing with spinal deformities were also analyzed.

## 2 Materials and methods

The study involved 116 (80 women and 36 men) professionally active Polish physiotherapists at the age of 24–62 years. The inclusion criterion was a professional physiotherapy license. The study was conducted between September 2022 and February 2023.

The research method was an online questionnaire designed by the authors. The questionnaire comprised closed-ended, open-ended, and mixed (closed- and open-ended) questions. The questionnaire was composed of 41 questions. The first part contained 15 questions about basic data related to the profession, while the second part contained 26 questions on scoliosis, physiotherapy treatment for IS and physical activities for persons with IS. The responses were coded as binary values (0 or 1). A correct answer received 1 point, and zero points were assigned for an incorrect answer or no answer. The questionnaire was developed based on the recommendations of the Society on Scoliosis Orthopedic and Rehabilitation Treatment (SOSORT) regarding the diagnosis and treatment of IS (1–3).

The second part of the questionnaire was divided into three sections. Section 1 was designed to verify the respondents’ knowledge about the characteristics of IS (maximum number of points that can be obtained was 8). Section 2 assessed the physiotherapists’ knowledge about the diagnosis and treatment of patients with IS (maximum number of points that can be obtained was 35). Section 3 evaluated the respondents’ knowledge about the recommended physical activities for subjects with IS (maximum number of points that can be obtained was 13).

In the questionnaire, the respondents’ knowledge was assessed on the following percent scale:

- 100–90% of correct answers – full knowledge (≥50 points obtained in the entire questionnaire; section 1: >7 points, section 2: ≥32 points, section 3: ≥12 points),
- 89–60% of correct answers – good knowledge (34–49 points obtained in the entire questionnaire, section 1: 5–7 points; section 2: 21–31 points; section 3: 8–11 points),
- 59–50% of correct answers – moderate knowledge (28–33 points obtained in the entire questionnaire; section 1: 4 points; section 2: 18–20 points; section 3: 6–7 points),
- Less than 50% of correct answers – poor knowledge (≤ 27 points obtained in the entire questionnaire; section 1: ≤ 3 points; section 2: ≤ 17 points; section 3: ≤ 5 points).

The statistical analysis was performed using Statistica 13.3 (StatSoft, Poland) and included the descriptive statistics. Normal distribution was assessed with the use of the Shapiro–Wilk test. Kruskall-Wallis test were used to assess the differences between independent groups. The Tukey test for unequal sample sizes was used as a *post-hoc*. The value *p* < 0.05 was adopted as the level of significance.

## 3 Results

The physiotherapists participating in the study correctly answered from 60 to 89% of the questions in the questionnaire which meant their good level of knowledge on the characteristics of idiopathic scoliosis, its diagnosis and treatment, and physical activity for patients with IS.

The overall mean score obtained by physiotherapists was 39.5 ± 6.7 points (79%). Regarding characteristics of IS (section 1) the respondents obtained 7.0 ± 1.1 points (87.5%) which indicate good knowledge about this topic. The mean score in the second section of the questionnaire was 23.8 ± 5.3 points (68%), which indicates that the respondents had a good knowledge about diagnosis and treatment of patients with IS. The surveyed subjects also had a good knowledge of the recommended physical activities for subjects with IS, and they scored an average of 7.8 ± 1.7 points (60%) in the third section of the questionnaire (Table 1).

**Table 1:** The level of knowledge on the characteristics of idiopathic scoliosis, its diagnosis and treatment, and physical activity for patients with IS.

	Mean ± SD	%	Knowledge
Entire questionnaire	39,5 ± 6,7	79.0	Good
Characteristics/definition	7,0 ± 1,1	87.5	Good
Diagnosis and treatment	23,8 ± 5,3	68.0	Good
Physical activity	7,8 ± 1,7	60.0	Good

Postgraduate courses influenced the knowledge of Polish physiotherapists regarding characteristic of IS (*p* = 0.003). Physiotherapists who completed courses based on physiotherapy recommended by SOSORT [so-called Physiotherapeutic Scoliosis Specific Exercises, PSSE (1)] demonstrated a higher level of knowledge compared to those without postgraduate courses (7.8 ± 0.4 vs. 6.2 ± 2.1; *p* = 0.018, 97.5% vs. 77.5%, respectively). The respondents without post-graduate courses had a good knowledge of IS characteristics, whereas physiotherapists who had completed post-graduate courses had full knowledge about the topic (Table 2).

**Table 2:** Influence of post-graduate courses on the physiotherapists’ knowledge about the characteristics of idiopathic scoliosis, diagnosis and treatment and recommended physical activities for patients with idiopathic scoliosis.

	Characteristics of IS		Diagnosis and treatment		Physical activities	
	Mean ± SD	Min–Max	Mean ± SD	Min–Max	Mean ± SD	Min–Max
No post-graduate courses* (*n* = 9)	6.2 ± 2.1	2.0–8.0	20.2 ± 6.7	9.0–29.0	7.6 ± 1.7	5.0–11.0
Courses on PSSE* (*n* = 10)	7.8 ± 0.4	7.0–8.0	26.6 ± 3.2	7.0–31.0	8.0 ± 1.9	5.0–11.0
Courses other than PSSE (*n* = 64)	6.8 ± 1.0	4.0–8.0	23.7 ± 5.6	11.0–33.0	7.7 ± 1.8	3.0–12.0
PSSE and other courses (*n* = 33)	7.3 ± 1.0	4.0–8.0	24.2 ± 4.4	17.0–31.0	7.8 ± 1.5	5.0–10.0
	*p* = 0.003 *Post-hoc: *p* = 0.018		*p* = 0.132		*p* = 0.910	

PSSE, Physiotherapeutic Scoliosis Specific Exercises.The “*” symbol shows between which groups the *post hoc* test revealed significant differences.

Despite the insignificant differences between post-graduate courses on PSSE and the respondents’ knowledge about the diagnosis and treatment of IS and the recommended physical activities for patients with IS, the surveyed subjects differed in their knowledge levels. The respondents who had completed post-graduate courses had a good knowledge of the above topics (diagnosis and treatment: 26.6 ± 3.2; 76%, physical activities: 8.0 ± 1.9; 61.5%), whereas physiotherapists without post-graduate training presented a moderate knowledge of both topics (diagnosis and treatment: 20.2 ± 6.7, 57.7%; physical activities: 7.6 ± 1.7, 58.4%) (Table 2).

The workplace influenced the knowledge of Polish physiotherapists regarding characteristics of IS (*p* = 0.036). Physiotherapists working in private healthcare facilities and working in both university and the private sector were characterized by full knowledge about this topic (7.5 ± 0.7, 7.3 ± 0.9; 93.7, 91.2%, respectively). The lowest mean score (6.0 ± 1.5; 75%) denoting good knowledge was noted in the group of physiotherapists working in private sector. Although the result of the Kruskall-Wallis test showed statistical significance, the *post-hoc* test did not reveal significant differences between the groups (Table 3).

**Table 3:** Influence of the place of employment on the physiotherapists’ knowledge about the characteristics of idiopathic scoliosis, diagnosis and treatment and recommended physical activities for patients with idiopathic scoliosis.

	Characteristics of IS		Diagnosis and treatment		Physical activities	
	Mean ± SD	Min–Max	Mean ± SD	Min–Max	Mean ± SD	Min–Max
Hospital (*n* = 14)*	6.7 ± 1.1	5.0–8.0	24.1 ± 5.9	15.0–33.0	6.6 ± 1.5	3.0–9.0
Private individual physiotherapy practice (*n* = 32)*	6.7 ± 1,3	2.0–8.0	23.3 ± 5.8	11.0–33.0	8.4 ± 1.8	5.0–12.0
Private healthcare facility (*n* = 34)	7.5 ± 0.7	5.0–8.0	24.6 ± 4.5	17.0–32.0	7.7 ± 1.6	4.0–11.0
Private sector (*n* = 7)	6.0 ± 1.5	4.0–8.0	23.6 ± 3.8	18.0–28.0	6.4 ± 1.7	5.0–10.0
Hospital and the private sector (*n* = 6)	6.2 ± 1,5	4.0–8.0	23.5 ± 6.3	14.0–31.0	7.3 ± 2.2	5.0–11.0
University and the private sector (*n* = 13)	7.3 ± 0.9	5.0–8.0	24.8 ± 4.2	19.0–31.0	7.7 ± 1.4	5.0–9.0
Other facilities (*n* = 9)	6.8 ± 1.2	5.0–8.0	20.8 ± 7.2	9.0–30.0	8.7 ± 1.5	6.0–11.0
	*p* = 0.036 *Post-hoc*: *p* > 0.05		*p* = 0.827		*p* = 0.008 *Post-hoc: *p* = 0.058	

Private sector, private individual physiotherapy practice and private healthcare facility.

Other facilities, sports clubs, gyms, schools, counseling and educational centers.The “*” symbol shows between which groups the *post hoc* test revealed significant differences.

Regarding the diagnosis and treatment of IS there were no significant differences between workplace and the respondents’ knowledge (Table 3).

The workplace influenced the knowledge of Polish physiotherapists regarding recommended physical activities for patients with IS (*p* = 0.008). The physiotherapists who ran the private individual physiotherapy practice demonstrated a higher level of knowledge compared to those working in a hospital (8.4 ± 1.8 vs. 6.6 ± 1.5; 64.6% vs. 52.3%, *p* = 0.058). Hospital employees had moderate knowledge (6.6 ± 1.5), whereas physiotherapists running a private practice had good knowledge (8.4 ± 1.8). The highest mean score (8.7 ± 1.5, 66.9%) denoting good knowledge was noted in the group of physiotherapists working in other facilities, including sports clubs, gyms, schools, and counseling and educational centers (Table 3).

There were no significant differences between academic degree and the respondents’ knowledge about the characteristics of idiopathic scoliosis as well as its diagnosis and treatment (Table 4).

**Table 4:** Influence of academic degree on the physiotherapists’ knowledge about the characteristics of idiopathic scoliosis, diagnosis and treatment and recommended physical activities for patients with idiopathic scoliosis.

	Characteristics of IS		Diagnosis and treatment		Physical activities	
	Mean ± SD	Min–Max	Mean ± SD	Min–Max	Mean ± SD	Min–Max
No academic degree (*n* = 107)	6.9 ± 1.1	2.0–8.0	23.7 ± 5.4	9.0–33.0	7.7 ± 1.7	3.0–12.0
PhD degree (*n* = 8)	7.6 ± 0.7	6.0–8.0	25.8 ± 3.6	20.0–29.0	8.8 ± 1.0	7.0–10.0
University professor (*n* = 1)	8.0 ± (not applicable)	8.0–8.0	25.0 ± (not applicable)	25.0–25.0	5.0 ± (not applicable)	5.0–5.0
	*p* = 0,085		*p* = 0,554		*p* = 0.05 *Post-hoc*: *p* > 0.05	

Regarding the recommended physical activities for patients with IS the academic degree influenced the knowledge of Polish physiotherapists (*p* = 0.05). The respondents with a PhD degree demonstrated the highest level of knowledge about the recommended physical activities for patients with IS in comparison to respondents without an academic degree (good knowledge, 8.8 ± 1.0, 67.7% vs. moderate knowledge 7.7 ± 1.7 points, 59.2%, respectively). A respondent with the title of a university professor received a score of 5.0 points, which indicates poor knowledge about the recommended physical activities for patients with IS. Although the result of the Kruskall-Wallis test showed statistical significance, the *post-hoc* test did not reveal significant differences between the groups (Table 4).

There were no significant differences between work experience and the respondents’ knowledge about the characteristics of idiopathic scoliosis, as well as diagnosis, treatment and recommended physical activities (Table 5).

**Table 5:** Influence of work experience on the physiotherapists’ knowledge about the characteristics of idiopathic scoliosis, diagnosis and treatment and recommended physical activities for patients with idiopathic scoliosis.

	Characteristics of IS		Diagnosis and treatment		Physical activities	
	Mean ± SD	Min–Max	Mean ± SD	Min–Max	Mean ± SD	Min–Max
I do not treat patients with scoliosis or other spinal deformities (*n* = 45)	6.6 ± 1.3	2.0–8.0	22.9 ± 6.2	9.0–33.0	7.8 ± 1.8	3.0–11.0
Less than a year (*n* = 8)	6.9 ± 1.0	5.0–8.0	22.1 ± 5.6	11.0–29.0	8.5 ± 2.4	5.0–12.0
1–3 years (*n* = 21)	7.2 ± 0.8	5.0–8.0	25.2 ± 4.3	19.0–33.0	7.4 ± 1.7	5.0–10.0
4–5 years (*n* = 16)	7.4 ± 0.9	5.0–8.0	25.0 ± 4.8	14.0–32.0	7.7 ± 1.1	6.0–9.0
6–10 years (*n* = 12)	7.4 ± 0.7	6.0–8.0	21.8 ± 3.4	17.0–27.0	7.3 ± 1.0	6.0–9.0
11–15 years (*n* = 13)	7.1 ± 1.3	4.0–8.0	26.2 ± 4.2	18.0–31.0	8.2 ± 2.0	5.0–11.0
16–20 years (*n* = 0)	–	–	–	–	–	–
21 years and more (*n* = 1)	8.0 ± (−)	8.0–8.0	27.0 ± (−)	27.0–27.0	9.0 ± (−)	9.0–9.0
	*p* = 0.063		*p* = 0.153		*p* = 0.514	

There were no significant differences between education level and the respondents’ knowledge about the characteristics of idiopathic scoliosis, diagnosis, treatment and recommended physical activities for patients with IS (Table 6).

**Table 6:** Influence of education on the physiotherapists’ knowledge about the characteristics of idiopathic scoliosis, diagnosis and treatment and recommended physical activities for patients with idiopathic scoliosis.

	Characteristics of IS		Diagnosis and treatment		Physical activities	
	Mean ± SD	Min–Max	Mean ± SD	Min–Max	Mean ± SD	Min–Max
Bachelor’s degree (*n* = 2)	5.0 ± 1.4	4.0–6.0	22.0 ± 5.7	18.0–26.0	7.0 ± 2.8	5.0–9.0
Master’s degree (*n* = 111)	7.0 ± 1.1	2.0–8.0	23.8 ± 5.3	9.0–33.0	7.7 ± 1.7	3.0–12.0
Master’s degree with specialization (*n* = 3)	7.3 ± 0.6	7.0–8.0	26.7 ± 4.0	22.0–29.0	8.3 ± 1.5	7.0–10.0
	*p* = 0.110		*p* = 0.496		*p* = 0.740	

Taking into account the fact that only one of the respondents is a member of association dealing with spinal deformities (SOSORT), only descriptive statistics were possible. The respondent declaring membership had full knowledge of the characteristics of IS and obtained a score of 8.0 points (100%). For diagnosis and treatment the respondent obtained 21.0 points (60%, good knowledge), while for physical activity the score was 5.0 (38.4%, poor knowledge) (Table 7).

**Table 7:** Influence of membership in associations dealing with spinal deformities on the physiotherapists’ knowledge about the characteristics of idiopathic scoliosis, diagnosis and treatment and recommended physical activities for patients with idiopathic scoliosis.

	Characteristics of IS		Diagnosis and treatment		Physical activities	
	Mean ± SD	Min–Max	Mean ± SD	Min–Max	Mean ± SD	Min–Max
Membership (*n* = 1)	8.0 ± (−)	8.0–8.0	21.0 ± (−)	21.0–21.0	5.0 ± (−)	5.0–5.0
No membership (*n* = 115)	7.0 ± 1,2	2.0–8.0	23.9 ± 5.3	9.0–33.0	7.8 ± 1.7	3.0–12.0

## 4 Discussion

The aim of the study was to assess physiotherapists’ knowledge about idiopathic scoliosis, its diagnosis and treatment as well as recommended physical activity. Factors such as post-graduate courses, place of employment, academic degree, work experience, education and membership in associations dealing with spinal deformities that could influence the respondents’ knowledge were also analyzed.

The study demonstrated that the surveyed physiotherapists had a good knowledge of IS. Key factors improving the level of physiotherapists knowledge about scoliosis were post-graduate courses (courses on Physiotherapeutic Scoliosis Specific Exercises), place of employment (private individual physiotherapy practice) and academic degree.

Training in courses on physiotherapy recommended by SOSORT significantly improve physiotherapists’ knowledge about the characteristics of IS. Despite the absence of significant differences between the completion of courses based on the SOSORT guidelines and the respondents’ knowledge about the diagnosis and treatment of IS and the recommended physical activities for patients with IS, the surveyed physiotherapists differed in their knowledge levels. The respondents who had completed post-graduate courses had a good knowledge of the above topics, whereas the physiotherapists who had not participated in such training had a moderate knowledge of both topics.

It should be noted that respondents working in private healthcare facilities demonstrated the higher level of knowledge about the characteristics of IS. This observation could imply that the private healthcare facilities offer a more supportive environment for professional development. An interesting observation is that respondents working both in a university and in the private sector demonstrated the second highest mean score of knowledge about a characteristic of IS. Physiotherapists who are academic lecturers are probably more motivated to update and expand their knowledge to provide students with high-quality education. The study also demonstrated that the physiotherapists who ran the private individual physiotherapy practice demonstrated a higher level of knowledge about physical activities for patients with IS compared to those working in a hospital. Interestingly, physiotherapists working in sports clubs and gyms received the highest mean scores of knowledge about physical activities for patients with IS. The above could be attributed to the fact that this group of respondents had greater experience in selecting the most beneficial exercises for patients with IS.

An analysis examining the influence of an academic degree on the physiotherapists’ knowledge about the recommended physical activities for patients with IS revealed that respondents with a PhD degree were characterized by good knowledge. Surprisingly, a respondent with the title of a university professor had a poor knowledge of the recommended physical activities for patients with IS. The physiotherapist with the title of a university professor scored much better results in the part of the questionnaire concerning the characteristics of IS and the diagnosis and treatment of the condition, which could point to higher levels of theoretical than practical knowledge.

Black et al. (10) assessed 206 British physiotherapy students for their knowledge of IS. The respondents were evaluated for their knowledge about the definition, causes, development, epidemiology, diagnosis, and conservative management of IS. The study demonstrated that 88% of the surveyed students were not familiar with the correct definition of IS. Half of the respondents (52%) correctly answered that IS is a condition with an unknown etiology, and 79% of the students recognized when IS is likely to develop. Only 24% of the students correctly answered the question on the prevalence of IS. In turn, only 12% of the respondents were familiar with the criteria for IS diagnosis, and 93% were unable to identify the appropriate treatment. More than half of the surveyed students (54%) correctly identified situations in which bracing is recommended in IS treatment. In the cited study, only 7% of the respondents answered more than 50% of the questions correctly (10).

A comparison of the results scored by physiotherapy students in the UK and Polish physiotherapists revealed significant differences in their knowledge about IS, in particular the methods of IS diagnosis and treatment. Polish respondents had a good knowledge of the above topics whereas very few British students gave correct answers (7% of the students answered more than 50% of the questions correctly). The lowest levels of knowledge were noted in the part of the questionnaire concerning the diagnosis (12% of the respondents gave correct answers) and treatment of IS (7% of the respondents gave correct answers). This difference could be attributed to the fact that the British study was conducted on physiotherapy students, whereas the present study involved licensed and professionally active physiotherapists in Poland.

Ciażyński et al. (14) conducted a study at the Medical University of Silesia to evaluate Polish physiotherapy students’ knowledge about IS. The study involved 37 third-year physiotherapy students aged 22–25 years. Eighty one percent of the respondents were familiar with the definition of IS. All respondents correctly answered the question on the etiology of IS, but the question on the diagnosis of IS was answered correctly by 62.2% of the surveyed subjects. The respondents were familiar with the following methods of conservative treatment: Lehnert-Schroth-Weiss method (94.6%), Klapp’s method (91.9%), Majoch’s method (89.2%), and Dobosiewicz’s method (78.4%). According to the surveyed students, the optimal types of physical activity for patients with IS were swimming (94.6%), yoga (73.0%), and martial arts (32.4%). The authors of the study concluded that physiotherapy students were generally characterized by insufficient knowledge about IS (14).

A comparison of the results reported in the survey of physiotherapy students and our findings revealed certain similarities in the administered questions. Both questionnaires addressed the characteristics of IS, diagnosis and treatment methods, and the recommended physical activities for patients with IS. In a study by Ciażyński et al. (14), the respondents were characterized by unsatisfactory levels of knowledge (14), whereas the physiotherapists surveyed in the current study had a good knowledge of these topics. However, the results of the studies cannot be directly compared due to differences in the administered questionnaires and sample size [the population examined by Ciażyński et al. (14) was three times smaller]. In addition, the cited survey analyzed physiotherapy students, whereas the current study was conducted on professionally active physiotherapists. What is more, in our study much more factors was took into consideration (post-graduate courses, work experience, and place of employment), which makes impossible to compare results directly.

Another study assessing physiotherapists’ knowledge about IS was conducted by du Toit et al. (12) in South Africa (*n* = 223). The respondents were surveyed with the use of an online questionnaire that was developed based on the SOSORT guidelines and contained 13 questions on the characteristics of IS, diagnosis and treatment methods involving physiotherapy and bracing. One-third (33.6%) of the respondents correctly answered more than 50% of the questions, and only 16.5% of the surveyed physiotherapists correctly answered more than 70% of the questions. The respondents’ knowledge about the characteristics of IS also differed depending on the question. The surveyed subjects were highly familiar with the etiology of the condition, but not with the definition or the prevalence of IS. The respondents were also characterized by low levels of knowledge about the diagnosis and treatment of IS (12).

The Polish physiotherapists examined in the present study were more knowledgeable about the diagnosis and treatment of IS than the group analyzed in South Africa. The observed differences in knowledge could be attributed to variations in physiotherapy programs across countries. The physiotherapists who had completed study programs based on the SOSORT guidelines received higher scores. However, physiotherapists differ in their ability to remember and integrate the acquired knowledge, as well as their adherence to the SOSORT guidelines in daily practice.

Akgül et al. (13) examined a large group of physiotherapist from Turkey, including 497 physiotherapy students and 649 professionally active physiotherapists. The questionnaire contained eight questions testing the respondents’ knowledge on the definition, causes, development, prevalence, diagnosis, and treatment of IS, physical activities for patients with IS, and indications for bracing, as well as three multiple-choice questions analyzing the respondents’ opinions on exercises for patients with IS. The findings point to a knowledge gap between physiotherapy students and practising physiotherapists. Physiotherapists scored higher results in all analyzed categories, which could be attributed to greater work experience. It should also be noted that 13% of the surveyed physiotherapists had participated in post-graduate training courses, which enabled them to expand their knowledge about IS and could contribute to their better results during the survey. This observation was validated by the results of the present study which revealed that post-graduate training on specific physiotherapy methods increased the respondents’ knowledge about the characteristics of IS (13).

A comparison of the results noted in the Turkish study and the current study revealed differences in the respondents’ knowledge about the characteristics of IS, diagnosis and conservative management. Turkish researchers concluded that the surveyed physiotherapists and physiotherapy students had insufficient knowledge. In contrast, the results of the present study demonstrated that Polish physiotherapists have higher levels of knowledge about IS.

### 4.1 Limitation of the study

The size of the analyzed sample (*n* = 116) was a limitation of the study. However, it should be noted that the present study examined the largest group of Polish physiotherapists to date. The second limitation of the study was the unequal size of the subgroups (e.g., education: Bachelor’s degree, Master’s degree, Master’s degree with specialization). The study group included physiotherapists who had different education systems - two-stage and one-stage, which may influenced the size of the bachelor group. Geographical differences in the origin of the physiotherapists and the age differences of physiotherapists can be considered as a direction for further research.

### 4.2 Strengths of the study

The main strength of this study was an extensive survey questionnaire (designed based on the SOSORT guidelines) which, unlike in the referenced studies, supported a multifaceted analysis of the physiotherapists’ knowledge about IS. The study demonstrated that post-graduate training, place of employment, and academic degree exert the greatest influence on the physiotherapists’ knowledge. The online questionnaire was accessible to physiotherapists in all Polish regions, including respondents who do not work only with IS patients. The dissemination of data obtained from this and future research may allow to propose participation in courses based on SOSORT (Society on Scoliosis Orthopaedic and Rehabilitation Treatment) guidelines, which will expand the knowledge of physiotherapists about the characteristics, diagnosis and treatment of idiopathic scoliosis.

## 5 Conclusion

Polish physiotherapists have a good knowledge about the diagnosis, treatment and physical activity of patients with idiopathic scoliosis. Completion of post-graduate courses (courses based on physiotherapy recommended by SOSORT), place of employment (private individual physiotherapy practice) and academic degree influence the physiotherapists’ knowledge about idiopathic scoliosis. Work experience and education have no influence on the physiotherapists’ knowledge about idiopathic scoliosis.

## Data Availability

The original contributions presented in the study are included in the article/Supplementary material, further inquiries can be directed to the corresponding author.
