# Supplementary material for: Physiotherapists’ knowledge about the diagnosis, treatment and physical activity of patients with idiopathic scoliosis
Source: Front Med (Lausanne). 2024 Oct 10;11:1414709. doi: 10.3389/fmed.2024.1414709 (PMC11500194; doi:10.3389/fmed.2024.1414709)
Supplement: Supplementary file 1 [file Data_Sheet_1.PDF]

Supplementary Material

Knowledge about adolescent idiopathic scoliosis and its physiotherapy management - questionnaire

Date:………..

Choose one answer for the following questions apart from the questions where is other option

General informations

1. In what Country do you practice Physiotherapy? ....

2. In what City do you practice Physiotherapy? ....

3. How big is the population in the City where do you practice Physiotherapy? ....

4. Gender

a. Male
b. Female

5. Age (years): ....

6. In what year did you obtain your Physiotherapy qualification? ....

7. Are you?
a. Bachelor
b. Masters of Physiotherapy
c. Master of Physiotherapy with specialization
d. PhD or Professional Doctorate
e. Doctor of physical therapy, DPT
f. Other

8. Your scientific title:
a. Master of Physiotherapy
b. PhD
c. Assistant Professor
d. Associate Professor
e. Full Professor
f. Other
g. None

9. What is your place of work? (you can choose more than one answer)
a. Hospital
b. Private office
c. Private clinic
d. Sanatorium
e. Sport club
f. Fitness center
g. University
h. Other

10. Circle the post-gradual courses you have completed (you can choose more than one answer)
a. Manual therapy
b. McKenzie Method
c. International Schroth 3D Scoliosis Therapy
d. Side-shift
e. Functional Indivivdual Therapy of Scoliosis (FITS)
f. DoboMed / Dobosiewicz method
g. Scientific Exercise Approach to Scoliosis (SEAS)
h. Barcelona Scoliosis Physical Therapy School (BSPTS)
i. Lyon Method
j. NDT Bobath
k. Proprioceptive Neuromuscular Facilitation (PNF)
l. Vojta method
m. Schroth Best Practice / Lehnert-Schroth-Weiss
n. Ostheopathy
o. Chiropractic
p. Klapp method
q. Other ....

11. Circle the post-gradual courses you use in your practice (you can choose more than one answer):
a. Manual therapy
b. McKenzie Method
c. International Schroth 3D Scoliosis Therapy
d. Schroth Best Practice / Lehnert-Schroth-Weiss
e. Side-shift
f. Functional Indivivdual Therapy of Scoliosis (FITS)
g. DoboMed / Dobosiewicz method
h. Scientific Exercise Approach to Scoliosis (SEAS)
i. Barcelona Scoliosis Physical Therapy School (BSPTS)
j. Lyon Method
k. NDT Bobath
l. Proprioceptive Neuromuscular Facilitation (PNF)
m. Vojta method
n. Osteopathy
o. Chiropractic
p. Klapp method
q. Other

12. What type of patients do you see most? (you can choose more than one answer)
a. Infantile physiotherapy
b. Faults of body posture
c. Spinal deformities (eg. idiopathic scoliosis, Scheuermann disease)
d. Injuries
e. Back and peripheral joints pain
f. Neurological physiotherapy – children (eg. Cerebral palsy)
g. Neurological physiotherapy – adults (eg. Stroke)
h. Cardiology
i. Pulmonology
j. Sport physiotherapy
k. Fitness
l. Other ....

13. Are you a member of societies related to spinal deformities? (you can choose more than one answer)
a. Society on Scoliosis Orthopaedic and Rehabilitation Treatment, SOSORT
b. Scoliosis Research Society, SRS
c. International Research Society of Spinal Deformities, IRSSD
d. Other ....
e. I'm not a member of societies related to spinal deformities

14. For how many years have you treating patients with scoliosis and other spinal deformities?
a. I do not treat patients with scoliosis and other spinal deformities
b. less than 1 year
c. 1-3 years
d. 4-5 years
e. 6-10 years
f. 11-15 years
g. 16-20 years
h. 21 and more years

15. How many (average) patients with idiophatic scoliosis do you treat every week?
....

Questions related to idiopathic scoliosis and its management using physiotherapy

16. What is, according your opinion, idiopathic scoliosis?
a. Deformity of the spine causes by inequal length of lower limbs
b. Lateral curvature of the spine
c. A two-dimensional torsional deformity of the spine with unknown etiology
d. A three-dimensional torsional deformity of the spine with unknown etiology
e. I'm not sure

17. What causes idiopathic scoliosis?
a. It is caused by congenital, vertebral or rib malformation, and secondary to a variety of systemic or neuromuscular disorders
b. Idiopathic scoliosis is an unknown disorder that can be attributed to a malformation of the spine during week three to six in utero

c. Idiopathic scoliosis is a structural scoliosis of the spine for which no specific cause can be established
d. Idiopathic scoliosis has a multifactorial etiology that consists of shortening of the one of the lower limbs, increase in paraspinal muscle tone, or a malformation of the thoracic cage
e. I'm not sure

18. When does idiopathic scoliosis commonly develop?
a. Idiopathic scoliosis develops in adulthood between the ranges of 35 years of age and older
b. Development of idiopathic scoliosis is attributed to a malformation of the spine during week three to six in utero. (Wiki Causes)
c. Idiopathic scoliosis may develop at any time during childhood and adolescence
d. Development of idiopathic scoliosis is a compensatory disorder that is a result of a traumatic injury or disease
e. I'm not sure

19. How prevalent is idiopathic scoliosis among patients with structural scoliosis?
a. Approximately 20% of cases are idiopathic scoliosis
b. Approximately 60% of cases are idiopathic scoliosis
c. Approximately 80% of cases are idiopathic scoliosis
d. Approximately 40% of cases are idiopathic scoliosis
e. I'm not sure

20. How is the diagnosis of idiopathic scoliosis commonly confirmed?
a. A Cobb angle is 20° or greater confirmed by X-rays
b. The patient presents with a rib hump and a lateral curvature in the spine confirmed by X-rays
c. The patient presents with asymmetrical iliac crest levels, 20° Cobb angle, and lateral curvature in the spine confirmed by X-rays
d. The Cobb angle is ≥ 10° and axial rotation can be recognised and confirmed by X-rays
e. I'm not sure

21. What are the consequences of idiopathic scoliosis? (you can choose more than one answer)
a. Back pain
b. Limited physical capacity due to pulmonary restriction
c. Worsening of the cosmetic aspect of the deformity (aesthetics)
d. Worsening of the quality of life
e. Limited ability to participate in physical education classes
f. Other ....

22. What is the aim of the treatment of idiopathic scoliosis? (you can choose more than one answer)
a. To stop a progression
b. To equalize length of the lower limbs
c. To improve an aesthetics
d. To improve the quality of life
e. To improve physical capacity and pulmonary parameters
f. To reduce/prevent back pain
g. To strength back muscles

h. To improve core stability
i. To strength abdominal muscles
j. To avoid surgery treatment
k. Other ....

23. What type of exercises and physical activities is not recommended for the treatment of adolescent idiopathic scoliosis? (you can choose more than one answer)
a. Exercises aimed at extension of the trunk
b. Asymmetrical breath exercises
c. Exercises aimed at core stability
d. Stretching
e. Swimming
f. Running
g. Gymnastics
h. Ballet
i. Other ....

24. What type of conservative treatment is not recommended for the adolescent idiopathic scoliosis? (you can choose more than one answer)
a. Physiotherapy
b. Osteopathy
c. Chiropractic
d. Manual therapy
e. Dry needling
f. Massage
g. Other ....

25. When is physiotherapy recommended for patients with idiopathic scoliosis as the primary treatment?
a. Scoliosis with Cobb angle value between 10-40˚
b. Scoliosis with Cobb angle value between 10-25˚
c. scoliosis with Cobb angle value between 25-40˚
d. physiotherapy should be the base of the treatment regardless of the Cobb angle value
e. I'm not sure

26. The treatment of idiopathic scoliosis using physiotherapeutic exercises is based on:
f. Stretching the concave side of the primary curve and strengthening the convex side of the primary curve in the spine
g. General physical activity like swimming or biking
h. Education, three-dimensional active self-correction, stabilization of the corrected posture, maintaining the corrected posture in activities of daily living
i. Education, three-dimensional active self-correction, maintaining the corrected posture in activities of daily living
j. Physiotherapy should be not used in the treatment of idiopathic scoliosis
k. I'm not sure

27. When is bracing recommended for patients with idiopathic scoliosis?
l. Patients that present with a primary curve between the ranges of 5°-10° Cobb angle should be recommended for scoliosis bracing

m. Bracing is recommended for patients that have been diagnosed with functional scoliosis that is secondary to a leg length discrepancy of 6mm or greater
n. Patients that present with a primary curve that is 45° Cobb angle or higher should be recommended for scoliosis bracing
o. Bracing is recommended for patients with a 25° (±5) Cobb angle that have an elevated risk of progressing
p. I'm not sure

28. What physical activity do you think would be beneficial to patients with idiopathic scoliosis? (you can choose more than one answer)
q. Swimming
r. Yoga
s. Martial Arts
t. Jogging
u. Pilates
v. Gymnastic
w. Other ....
x. I'm not sure

29. What physical activity do you think would be may be harmful to patients with idiopathic scoliosis? (you can choose more than one answer)
y. Gymnastics
z. Ballet Dancing
aa. Martial Arts
bb. Cycling
cc. Gymnastic
dd. Other ....
ee. I'm not sure

30. What is the aim of general physical activity for children with idiopathic scoliosis (eg. Physical education classes, running, playing football, swimming)? (you can choose more than one answer)
a. To stop progression of the scoliosis
b. To improve the aesthetics disturbed by scoliosis
c. To improve quality of life
d. To improve physical capacity
e. To improve the ability to perform exercises with higher level of intensity
f. To improve the neuromuscular coordination
g. A child with idiopathic scoliosis should not participate in general physical activity
h. Other…..

31. What Societies (local and international) are focused on the conservative treatment of idiopathic scoliosis?………..

32. According to evidence based research, what has been proven to be the most effective form of conservative management in idiopathic scoliosis? (you can choose more than one answer)
a. Sport activities
b. Kinesitherapy
c. Physiotherapeutic scoliosis-specific exercises

d. Manual therapy
e. Chiropractic
f. Osteopathy
g. Bracing
h. Other ....
i. None
j. I'm not sure

33. What is the aim of physiotherapy during brace treatment? (you can choose more than one answer)
a. To prepare soft tissues before bracing
b. To educate of the patient about the role of bracing
c. The verify the time of wearing brace
d. To teach how to perform exercises in-brace
e. To teach how to perform exercises without brace
f. During bracing the physiotherapy is unnecessary
g. To stabilize the posture during removing of the brace
h. I'm not sure

34. What is the aim of physiotherapy in surgery treatment? (you can choose more than one answer)
a. To prepare soft tissues before surgery
b. Neuromobilization
c. The stabilize the spine in the areas beside the spondylodesis
d. To improve the cosmetic aspect of the trunk
e. To improve the rib hump
f. To teach ergonomics
g. Before and after surgery physiotherapy is unnecessary
h. To improve the mobility of the spine after surgery
i. To improve mobility of the hip joints
j. Other ……………………………………………………………………………………
k. I'm not sure

35. After surgical treatment usually a patient is able to fully return to sport activities:
a. After 3 months
b. After 6 months
c. After 12 months
d. Will never return to sport activities
e. I'm not sure

36. Would you feel confident evaluating idiopathic scoliosis using the Adam's forward bending test together with the Scoliometer?
a. Yes
b. No. why? ....
c. I'm not sure

37. Would you feel confident in providing educational support to a patient presenting with idiopathic scoliosis?
a. Yes
b. No

c. I'm not sure

38. Would you feel confident in the management of a patient with idiopathic scoliosis?
a. Yes
b. No
c. I'm not sure

39. Do you feel that scoliosis specific physiotherapy exercise intervention can be beneficial in the management of idiopathic scoliosis?
a. Yes
b. No
c. I'm not sure

40. A 10 years young girl, without menarche presents angle of trunk rotation 5˚. What is your recommendation?
a. You recognize idiopathic scoliosis
b. You will send a girl for an X-ray
c. You will ask a girl to return for control examination during next 6 months
d. You will say that there is no risk of scoliosis
e. I'm not sure

41. A 14 years young girl, with menarche for two years presents an angle of trunk rotation of 6˚. What is your recommendation?
a. You recognize idiopathic scoliosis
b. You will send a girl for an X-ray
c. You will ask a girl to return for control examination during next 6 months
d. You will say that there is no risk of scoliosis
e. I'm not sure
